# New York Academy of Medicine

**Published:** 1889-06

**Authors:** 


### NEW YORK ACADEMY OF MEDICINE.

Section on Neurology.

Landon Carter Gray, M.D., Chairman.

*Stated Meeting, April 12, 1889.*

NERVOUS SYMPTOMS ARISING FROM OVARIAN DISEASE, AND THEIR TREATMENT.

Dr. Charles L. Dana said that the term ovarian irritation is an uncertain one, and hardly correct, since the uterus and the tubes are appendages of the ovaries and may share in the trouble. The changes in the organ are anatomical and physiological. The most frequent one is chronic hyperæmia, which he says is the same thing as chronic ovaritis. To this condition are referred vasomotor disturbances, cardiac disorders, hysteria, epilepsy, mania, melancholia, chorea, neuralgic states, spinal irritation, sciatica, migraine, and irritability of the bladder and rectum. In his opinion chronic ovarian hyperæmia cannot produce any of the severe constitutional neuroses, and that removal of the ovaries does not, as a rule, remove grave neuroses, such as chorea, epilepsy, melancholia, and intractable neuralgias. In about one-half of such cases in women the ovaries are perfectly healthy, while in those whose ovaries are thoroughly diseased no such neurotic symptoms occur. He thought that many of Battey's cures were simply cases of convulsive hysteria which would have recovered under any treatment. Of ovarian mania, Battey reports four cases with one cure, and Goodell five cases with four cures, from removal of the ovaries. These were probably cases of hysterical insania in which, according to Clouston, the proportion of cures under any treatment is sixty per centum; upon the whole he thought that the ovaries by themselves occupy a very subordinate place in the production of neuroses.

Dr. W. Gill Wylie said that he was substantially in accord with the main points made by Dr. Dana. Imperfectly developed women are especially prone to have diseased nervous systems, and it is often very difficult to separate symptoms actually caused by diseased ovaries from those caused by an imperfectly developed nervous organism. He has also found that most cases of uterine or ovarian disease are complicated by at least an abnormal condition of the colon or rectum, and many brilliant cures of supposed uterine or ovarian disease are made by dilating the anus and thus removing reflex disturbances caused by anal pressure. Similar facts are true in cases of dilated and sacculated colon.

An inflamed ovary will cause reflex symptoms, especially for some days preceding menstruation, on account of the increased vascular tension existing at that time. An ovary imprisoned by a firm peritoneal exudation will cause unusual reflex symptoms and local pain, due to the resistance offered to the maturing ovum swelling under the peritoneal adhesions, or to the occluded Fallopian tube and abnormal ovulation resulting in cystic degeneration and the swelling cysts stretching the peritoneal adhesions. He has seen several cases where the reflex symptoms amounted almost to insanity, caused, as was shown when the abdomen was opened, by a thickening and hardening of the ovarian tissue, resulting in the production of a very great increase of the fibroid tissue, and, in some cases, of small nodules or fibroid tumors on or in the ovary. In this class of cases the patients suffer intense local pain, and just before menstruation become very irritable, and excitable to an extreme degree.

As to treatment, in all cases where the reflex symptoms or the nervous symptoms are marked, he invariably gets the advice of a neurologist before resorting to active treatment, so as to avoid the mistake of exciting and bringing to a climax some commencing or undeveloped disease of the nervous system. Then, before attempting to reach a positive diagnosis he makes it a rule to keep the patient for some weeks under his own observation, unless the symptoms demand immediate surgical interference. Also before deciding whether an ovary is enlarged or decreased, he thinks it wise to insist upon an examination of the patient under ether. If one can plainly make out disease of the ovaries, indicated by fixation or enlargement —that is, permanent enlargement, the organ being as large as a small hen's egg— and if the symptoms are not relieved or rendered latent by improving the general health of the patient, or by the use of means to improve the local circulation, such as counter-irritants, etc., then laparotomy should be considered, if the subjective symptoms warrant it.

But unless actual physical disease can be plainly made out by local examination under ether, he would not advise laparotomy until every other means, in the hands of several different physicians, have been faithfully tried. He said he was sure that in many cases where the ovaries have been removed, if the influence of imperfect development had been recognized, and a simple stimulating local treatment, combined with careful attention to improving the general condition, had been properly carried out and persisted in, the patients would have saved their ovaries and regained their health as completely, if not more completely, as by castration. In these cases he said he was accustomed to apply cotton pledgets soaked in a solution of boro-glyceride and glycerine twice a week, to dilate the uterine canal once a week between the menstrual periods, and make a stimulating intra-uterine application. In some cases he applied electricity, and in others put in a drainage plug for a week every three or four months, etc.

When there was distinct nervous disease he did not think much could be accomplished by the removal of the ovaries. Personally he had performed the operation in but five cases, and only one of the patients was cured, while three were not at all benefited.

Dr. B. Sachs said that in his own experience, in cases of true hysteria the symptoms always return after a longer or shorter period.

Dr. M. P. Jacobi said that it was her opinion that ovaritis never occurs as a primary disease, but is always associated with an endometritis of the fundus of the uterus, and she made the conservative remarks which are associated with such an opinion.

Dr. W. R. Birdsall agreed very fully with Dr. Wylie. He knew of patients who successively had their nasal septa straightened, their ocular muscles cut, their uteri cured of malpositions, and their bladders relieved of irritation. All of these methods of treatment had improved the condition of the patient for the time being, but scarcely had one set of symptoms been controlled when a new set would be developed. He referred to cases of general neuralgias not relieved by nerve resection, and of removal of ovaries without even temporary benefit, as showing that the removal of a painful organ does not necessarily cure a neuralgic pain long established. In conclusion, he said there are two extremes to this question, "but that in the future no doubt cases would occur in which removal of the ovaries would be attended with benefit."

Dr. H. T. Hanks agreed substantially with Dr. Wylie.

Dr. Boldt agreed with the general conservative opinion and added that as a result of his experience he thought that in cases in which the ovaries appear to be normal, no matter how severe the nervous symptoms may be, the removal of the ovaries will not afford relief. In some cases of nervous trouble the symptoms were decidedly worse after removal than they had been before, and he thought that the ovaries should never be removed until all other means of treatment had been exhausted.

